# Analytical Methods for Fluid Biomarkers in Alzheimer’s Disease from Discovery to Clinical Implementation

**DOI:** 10.3390/ijms27104518

**Published:** 2026-05-18

**Authors:** Luisa Agnello, Roberto Dominici, Caterina Maria Gambino, Concetta Scazzone, Marcello Ciaccio

**Affiliations:** 1 Department of Biomedicine, Neurosciences, and Advanced Diagnostics, Institute of Clinical Biochemistry, Clinical Molecular Medicine, and Clinical Laboratory Medicine, University of Palermo, 90127 Palermo, Italy; luisa.agnello@unipa.it (L.A.); caterinamaria.gambino@unipa.it (C.M.G.); concetta.scazzone@unipa.it (C.S.); 2 Department of Laboratory Medicine, University Hospital Paolo Giaccone, 90127 Palermo, Italy; 3 University Laboratory of Clinical Pathology and Toxicology, Pio XI Hospital, Desio, ASST Brianza, 20832 Desio, Italy; robertodominici65@gmail.com

**Keywords:** biomarkers, biofluids, Alzheimer’s disease, CSF, blood, FDA, discovery

## Abstract

Alzheimer’s disease (AD) is increasingly recognized as a biological continuum characterized by early neuropathological and molecular changes that precede the onset of clinical symptoms. Fluid biomarkers have transformed the diagnostic landscape by enabling the in vivo detection of core AD pathologies, particularly amyloid-β deposition and tau-related neurodegeneration. Despite the rapid expansion of candidate biomarkers, however, only a limited number have successfully translated into clinical practice. Discovery-phase approaches, primarily driven by mass spectrometry-based proteomics, enable the unbiased identification of novel biomarker candidates across multiple biological pathways. Research-phase methods, including immunoassays such as enzyme-linked immunosorbent assay (ELISA), electrochemiluminescence immunoassays (ECLIA), microfluidic platforms, and ultrasensitive technologies such as single-molecule array (SIMOA), support analytical and clinical validation in well-characterized cohorts. Clinical implementation has been advanced by fully automated platforms, including Lumipulse and Elecsys, which have obtained regulatory approval for cerebrospinal fluid biomarkers and, more recently, blood-based biomarkers. These developments represent a paradigm shift toward minimally invasive and scalable diagnostic strategies that may reduce dependence on neuroimaging techniques. Nevertheless, major challenges remain, including assay standardization, inter-platform variability, demonstration of clinical utility, and barriers to widespread clinical adoption. This review provides a comprehensive overview of analytical methods used to measure AD fluid biomarkers in cerebrospinal fluid and plasma, structured according to the biomarker development pipeline from discovery to clinical implementation. Overall, the review highlights a fit-for-purpose approach to biomarker development and emphasizes the complementary roles of diverse analytical technologies across the different phases of biomarker translation.

## 1. Introduction

Alzheimer’s disease (AD) affects over 60% of the 57 million individuals with dementia worldwide, with prevalence projected to exceed 100 million by 2050 [1]. It is a multifactorial neurodegenerative disorder characterized by progressive cognitive decline and substantial biological heterogeneity. Epidemiological studies indicate a higher prevalence of AD in women compared with men, potentially reflecting differences in longevity, hormonal status, genetic susceptibility, and immune responses [2]. In addition to aging, several environmental and lifestyle-related factors have been associated with increased AD risk, including cardiovascular disease, diabetes, obesity, smoking, physical inactivity, air pollution exposure, and low educational attainment [3]. Genetic contributions also play a central role in AD pathogenesis [4]. While rare familial forms are associated with pathogenic variants in APP, PSEN1, and PSEN2, the APOE ε4 allele remains the strongest genetic risk factor for late-onset AD. Advances in genomics have further identified multiple susceptibility loci involved in lipid metabolism, immune regulation, endosomal trafficking, and microglial function, including TREM2, CLU, PICALM, and BIN1 [5]. Genetic testing approaches, ranging from targeted mutation analysis to genome-wide association studies and polygenic risk scoring, are increasingly being explored for risk stratification and precision medicine applications.

Definitive diagnosis historically required postmortem neuropathological examination demonstrating amyloid-β (Aβ) plaques and neurofibrillary tangles [6]. The International Working Group’s 2007 revision of diagnostic criteria represented a paradigm shift, proposing that AD diagnosis could be anchored around biomarker evidence rather than clinical presentation alone [7]. This biological characterization has since been refined, with the 2024 Alzheimer’s Association workgroup defining AD as a biological process beginning with neuropathologic change during the asymptomatic phase [8,9]. The essential neuropathologic features of AD, including β-amyloid plaques and neurofibrillary tangles, can be detected in vivo by fluid biomarkers [10]. Over the past 25 years, three core CSF biomarkers for AD have been identified and extensively validated: Aβ42, which is found at low concentrations in patients with AD due to cortical amyloid deposition; t-Tau at high concentrations due to cortical neuronal loss; and p-Tau at high concentrations, reflecting cortical tangle formation [11]. These biomarkers, defined as core AD biomarkers, have been incorporated into modern diagnostic research criteria and form the foundation of the biological definition of AD [12]. Beyond core biomarkers, several molecules have emerged as useful tools for supporting AD diagnosis. However, only a few of them have been implemented in clinical practice [13].

**Figure 1:**
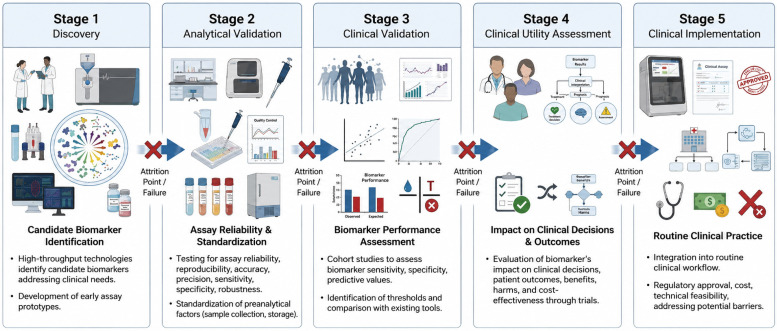
Pipeline for fluid biomarkers: from discovery to clinical implementation. The discovery phase includes high-throughput and unbiased omics approaches, particularly mass spectrometry-based proteomics, used to identify candidate biomarkers associated with amyloid pathology, tau pathology, neuroinflammation, vascular dysfunction, synaptic impairment, metabolic alterations, and AD co-pathologies. The research phase comprises targeted validation approaches, including conventional immunoassays (ELISA), high-sensitivity and ultrasensitive immunoassays, immunoprecipitation–mass spectrometry (IP-MS), targeted mass spectrometry, and multiplexed targeted proteomic platforms such as Olink and NULISA. The clinical phase illustrates standardized and automated platforms, including CLEIA, ECLIA, and targeted LC–MS/MS assays, designed for routine clinical implementation, patient stratification, disease monitoring, and therapeutic response assessment.

The translation of a biomarker from initial discovery to routine clinical use represents a complex, multistage process that requires rigorous scientific validation, regulatory oversight, and demonstration of clinical utility. Despite thousands of biomarkers being reported in the literature annually, very few progress to clinical implementation, a phenomenon often described as failures in the biomarker pipeline. As outlined in the Geneva roadmap, biomarker development follows a structured progression through five distinct phases [14] (Figure 1): (1) discovery; (2) analytical validation; (3) clinical validation; (4) clinical utility assessment; and (5) clinical implementation.

The discovery phase involves identifying candidate biomarkers that may address a specific clinical need. During this exploratory stage, researchers use various high-throughput technologies and analytical platforms to identify molecules, genes, or proteins that differ between disease and non-disease states. High-throughput technologies, particularly omics-based approaches such as proteomics, metabolomics, lipidomics, and transcriptomics, have greatly advanced the discovery of novel AD biomarkers. These unbiased analytical platforms enable large-scale characterization of molecular alterations across multiple biological pathways, providing insights into disease mechanisms and identifying potential biomarker candidates for further validation.

Well-controlled early assay prototypes are sufficient at this stage to determine which markers warrant further investigation. The discovery phase should ideally begin with consideration of clinical needs rather than being purely technology driven.

Analytical validation establishes that the biomarker assay performs reliably and reproducibly under controlled laboratory conditions. This phase requires the development of an analytically robust prototype with rigorous quality control to ensure results are valid across multiple research studies. Key analytical parameters that must be established include accuracy and precision, reproducibility across different laboratories (robustness), analytical sensitivity and specificity, matrix effects and identification of interfering substances, stability at various temperatures and storage conditions, dynamic range and limits of detection, and coefficient of variation to quantify analytical variability. Additionally, preanalytical factors, including sample collection, processing time, storage conditions, freeze–thaw cycles, fasting status, and circadian variations, must also be systematically evaluated and standardized through the development of standard operating procedures.

Clinical validation phase demonstrates that the biomarker accurately identifies, measures, or predicts the clinical or biological process of interest in the target population. This phase involves retrospective and longitudinal cohort studies to understand biomarker variability, identify appropriate thresholds, and confirm linkage to health outcomes. Clinical validation requires assessment of sensitivity and specificity in the intended use population, positive and negative predictive values, performance in relevant clinical subgroups, comparison with and combination with existing clinical tools and physician judgment. The biomarker must demonstrate that its distribution differs significantly between individuals who develop the outcome of interest versus those who do not, ideally established through prospective study designs.

Demonstrating clinical utility, the most critical yet challenging phase, requires evidence that using the biomarker leads to improved clinical outcomes compared to standard practice. Biomarker accuracy alone is insufficient; the test must reliably add to clinical judgment and result in more favorable outcomes for patients. Clinical utility assessment evaluates how the biomarker impacts clinical decisions (diagnostic workup, treatment selection), consequences of those decisions on patient outcomes (complications, quality of life, survival), both benefits from true results and harms from false results, and cost-effectiveness compared to current standards. Randomized controlled trials comparing outcomes with and without biomarker-guided management represent the gold standard for demonstrating clinical utility, though alternative study designs including decision curve analysis and comparative effectiveness studies may be employed.

The final phase involves widespread adoption into routine clinical practice. Successful implementation requires not only regulatory approval but also practical considerations, including cost, ease of sample collection, technical feasibility in diverse settings, and integration into clinical workflows. Implementation may be hindered even when clinical utility is demonstrated if the test is expensive, technically complex, or requires significant changes to practice culture.

The discovery phase prioritizes comprehensive, unbiased molecular profiling to identify novel candidates [15]. Research-phase methods emphasize analytical sensitivity and specificity to validate candidate biomarkers in well-characterized cohorts and establish their clinical usefulness. Clinical-phase methods focus on automation, standardization, and regulatory compliance to enable routine diagnostic use [16,17].

**Figure 2:**
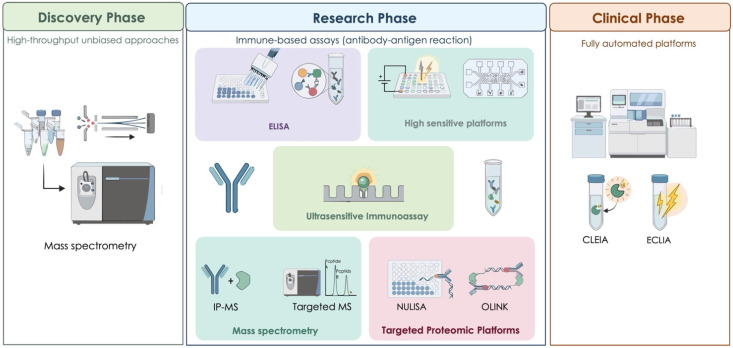
Analytical methods for measuring AD fluid biomarkers in each phase, from discovery to clinical implementation. In the discovery phase, high-throughput unbiased approaches, including mass spectrometry and RNA sequencing of fluid biomarkers, are used to identify candidate biomarkers. The research phase comprises immune-based assays exploiting antibody–antigen interactions, including ELISA, ultrasensitive immunoassays, high-sensitivity platforms, mass spectrometry-based targeted analyses (IP-MS and targeted MS), and targeted proteomic platforms such as NULISA and OLINK. In the clinical phase, fully automated platforms, including chemiluminescent enzyme immunoassays (CLEIA) and electrochemiluminescence immunoassays (ECLIA), are employed for routine clinical implementation and validation.

This review deals with the analytical methods for AD biomarker measurement organized by their primary application phase, with emphasis on the technical principles, performance characteristics, and appropriate use of each approach (Figure 2).

## 2. Discovery Phase

The discovery phase focuses on identifying novel biomarkers through high-throughput and unbiased approaches [18,19].

Mass spectrometry (MS)-based proteomics has become the core technology for comprehensive protein identification and quantification, enabling analysis of thousands of proteins in biological samples and serving as the primary platform for biomarker discovery [20,21].

The selection of biological fluids for biomarker discovery is guided by the balance between biological relevance, accessibility, analytical sensitivity, and clinical applicability. CSF is considered the gold-standard matrix for AD biomarker research because of its close anatomical and biochemical proximity to the central nervous system, allowing sensitive detection of core pathological processes such as amyloid-β deposition, tau pathology, neurodegeneration, and neuroinflammation. However, the invasive nature of lumbar puncture limits its widespread use in large-scale screening and longitudinal monitoring. In contrast, blood-based biomarkers provide a minimally invasive, scalable, and cost-effective alternative that is increasingly suitable for population-based studies, repeated sampling, and clinical implementation, despite the lower abundance of brain-derived proteins and potential peripheral confounding effects. Additional biofluids, including saliva, urine, and tears, are also being explored as accessible matrices that may capture peripheral or systemic manifestations associated with AD pathology and co-pathological processes. Together, these complementary biological matrices provide distinct yet overlapping information that supports multidimensional biomarker discovery and validation across the AD continuum.

By providing detailed information on protein identity, abundance, structure, and post-translational modifications, MS-based proteomics offers a powerful platform to identify candidate biomarkers associated with disease onset, progression, and therapeutic response.

The fundamental workflow of MS-based proteomics involves enzymatic digestion of proteins into peptides (typically using trypsin), separation by liquid chromatography (LC), and analysis by tandem mass spectrometry (LC-MS/MS) [22,23]. Proteins are first extracted from biological samples (tissues, body fluids), then subjected to reduction, alkylation, and enzymatic digestion to generate peptides. These peptides are separated by high-performance liquid chromatography and introduced into the mass spectrometer, where they are ionized, fragmented, and detected based on their mass-to-charge (*m*/*z*) ratios. The resulting mass spectra are searched against protein sequence databases or spectral libraries for protein identification [22]. Various downstream analyses including spectral counting, network analysis, and quantitative comparisons extract qualitative and quantitative information from the data.

MS-based approaches provide hypothesis-free discovery without requiring individual assay development for each target, casting the widest net toward potential biomarkers. The technology encompasses both untargeted discovery strategies (shotgun proteomics, data-independent acquisition) that provide unbiased proteome-wide profiling, and targeted approaches (SRM/MRM, PRM) that achieve the sensitivity necessary to detect and quantify low-abundance clinically important biomarkers.

Shotgun proteomics represents the most widely used global discovery strategy, providing an unbiased view of protein species without requiring individual assay development for each target. This approach casts the widest net toward potential biomarkers, covering thousands of proteins in a single experiment. The term “shotgun” reflects the comprehensive, unbiased nature of the approach, proteins are digested first, then peptides are sequenced, with protein identity inferred from the identified peptides (hence “bottom-up”) [24]. Once candidates are identified through shotgun approaches, they typically undergo validation using targeted proteomics methods (e.g., parallel reaction monitoring, SRM/MRM) that offer higher sensitivity and quantitative precision for selected protein panels [23,25]. This “fit-for-purpose” strategy leverages the complementary strengths of untargeted discovery and targeted validation approaches.

## 3. Research Phase

Research-phase methods bridge the gap between discovery and clinical implementation, emphasizing analytical validation and clinical performance assessment in well-characterized cohorts. These are immune-based assays, which rely on the antibody–antigen reaction. They can be classified as (i) Enzyme-Linked Immunosorbent Assays (ELISA), including manual ELISA platforms and multiplex bead-based immunoassays; (ii) highly sensitive platforms, including Electrochemiluminescence (ECL) Technology and Microfluidic ELISA Technology; (iii) Ultrasensitive Immunoassay Technologies, including Single Molecule Array (Simoa); (iv) mass spectrometry-based quantification, including Immunoprecipitation-Mass Spectrometry (IP-MS) and targeted Mass Spectrometry; (v) targeted proteomic platforms, including NULISA and OLINK.

### 3.1. Enzyme-Linked Immunosorbent Assays

ELISAs have served as the foundational analytical platform for CSF AD biomarker measurement over the past two decades, establishing the clinical utility of Aβ42, t-tau, and p-tau181 as diagnostic markers [26]. The ELISA methodology employs antibody pairs, capture and detection antibodies, that specifically bind the analyte of interest within microplate wells, forming sandwich immunocomplexes (capture antibody–analyte–detection antibody). The detection antibody is conjugated to an enzyme that catalyzes the conversion of a substrate to a product, generating fluorescence or colorimetric change proportional to analyte concentration, typically within the nanomolar to picomolar range.

The INNOTEST assay (Fujirebio Europe) represents the most widely utilized manual ELISA for the quantitative determination of CSF AD biomarkers, with extensive validation across research cohorts worldwide [27,28]. However, manual ELISA assays demonstrate significant inter-laboratory and intra-laboratory variability that substantially limits their clinical utility [29,30,31]. Based on the Alzheimer’s Association external quality control program (AAQC program), the peer group coefficient of variation in manual ELISA assays for CSF Aβ42 was unsatisfactory (>20%) [30]. A study investigating CSF variability found that reanalysis led to a change in biomarker classification (normal vs. abnormal) of 26% of subjects based on Aβ42, 10% based on t-tau, and 29% based on p-tau [29]. The changes in absolute biomarker concentrations were paralleled by similar changes in levels of internal control samples between different assay lots, identifying lot-to-lot variation as a major cause of intralaboratory variability. This variability has profound implications for clinical practice, as it precludes the establishment of universal cut-off values applicable across laboratories and necessitates laboratory-specific reference ranges.

The INNO-BIA AlzBio3 immunoassay utilizing Luminex xMAP technology represents an important advancement, enabling simultaneous measurement of multiple biomarkers from small sample volumes [32,33,34]. This bead-based sandwich immunoassay combined with flow cytometry overcomes the limitations of single-analyte ELISA by using dyed micro-beads coated with monoclonal antibodies, capable of detecting 100–500 immune markers in cerebrospinal fluid (CSF) from a single sample. The xMAP-Luminex platform offers several advantages, such as a wide dynamic range of ready-to-use calibrators, time savings for simultaneous analysis of three biomarkers in one analytical run, reduction in human error, potential reduced cost of reagents, and modest reduction in sample volume compared to conventional ELISA methodology [33]. Both ELISA and Luminex can be used to measure AD biomarker levels in CSF to support clinical diagnosis and predict progression from MCI to AD with similar accuracy; however, the assays’ output in absolute biomarker concentrations is remarkably different, necessitating platform-specific cutoff values [35,36]. The xMAP-Luminex platform remains primarily research use only (RUO) due to a combination of analytical validation challenges, regulatory pathway complexities, and standardization limitations inherent to multiplex immunoassay technologies [37,38].

In summary, while manual ELISA platforms established the foundational evidence for CSF AD biomarkers and remain widely used in research settings, their significant inter- and intra-laboratory variability limits clinical implementation. Multiplex bead-based immunoassays offer advantages in throughput and sample conservation with comparable diagnostic accuracy, though absolute values differ substantially from ELISA. Both platforms have been largely superseded by fully automated immunoassay systems (Lumipulse, Elecsys) that provide superior standardization, reproducibility, and regulatory compliance for clinical diagnostic use, as described in the clinical phase paragraph.

### 3.2. Highly Sensitive Immunoassay Technologies

High-sensitivity immunoassay platforms occupy an intermediate position between conventional ELISA (nanomolar to picomolar range) and ultrasensitive digital detection platforms (sub-pg/mL to fg/mL range), typically achieving detection limits in the low pg/mL range [39]. Two prominent technologies in this category are electrochemiluminescence (ECL) and microfluidic ELISA.

ECLIAs are antibody-based detection platforms that generate light through electrochemical reactions, offering enhanced sensitivity compared to conventional ELISA [40,41]. The technology works by labelling detection antibodies with electrochemically active molecules (typically ruthenium complexes such as [Ru(bpy)_3_]^2+^) that emit light when oxidized at an electrode surface in the presence of a coreactant (commonly tripropylamine) [42]. In the assay format, target proteins are captured by antibodies coated on electrode surfaces or magnetic beads, forming sandwich immunocomplexes with the ECL-labeled detection antibody. When voltage is applied to the electrode, the ruthenium label and coreactant undergo oxidation, generating excited-state ruthenium species that emit light at approximately 620 nm, with intensity proportional to the analyte concentration [43]. The Meso Scale Discovery (MSD) platform is the most widely used commercial ECLIA system, featuring multi-spot plates with carbon electrodes printed on the bottom of each well, enabling multiplexed detection of up to 10 different analytes simultaneously [44,45]. The platform utilizes SULFO-TAG™ labels (ruthenium-based compounds) conjugated to detection antibodies, which generate ECL signals when electrical stimulation is applied. MSD technology has demonstrated superior performance characteristics including extremely low detection limits (200 fmol/L), a dynamic range extending over six orders of magnitude (10^5^ to 10^6^), and the ability to use small molecule labels (approximately 1000 Da) that can be coupled to proteins or oligonucleotides without affecting immunoreactivity. While ECL immunoassays offer improved sensitivity over ELISA, they still face fundamental limitations inherent to all antibody-based methods, including susceptibility to antibody cross-reactivity, interference from heterophilic antibodies and autoantibodies, matrix effects in complex biological fluids, and the high-dose hook effect. The ECL signal can decrease significantly over time due to electrode surface fouling, though electrochemical regeneration treatments can restore initial signal strength. Additionally, like other immunoassays, ECL platforms achieve moderate diagnostic accuracies (62–79%) for detecting abnormal amyloid status compared to mass spectrometry methods (82–97%) and show lower correlations with CSF biomarkers [46]. Despite these limitations, ECL-based platforms like MSD represent a practical middle ground between conventional ELISA and more complex mass spectrometry approaches, offering improved sensitivity and multiplexing capability suitable for large-scale clinical validation studies.

Microfluidic ELISA technology is a technology platform that integrates the fundamental principles of enzyme-linked immunosorbent assay into microchannel chip systems, enabling miniaturized, rapid, and efficient analysis of protein biomarkers [47]. It retains the core immunoassay principle of conventional ELISA, utilizing antibody pairs (capture and detection antibodies) that specifically bind the analyte of interest to form sandwich immunocomplexes. However, the miniaturized format fundamentally alters reaction kinetics. The high surface-to-volume ratio inherent to microchannels accelerates antibody–antigen binding reactions, as diffusion distances are dramatically reduced compared to conventional 96-well plates [48,49]. This geometric advantage enables faster equilibration and more efficient capture of target analytes. The Ella (Simple Plex) platform (Bio-Techne) is the most widely adopted commercial microfluidic ELISA system for AD biomarker measurement. This automated platform employs disposable cartridges containing pre-loaded reagents in separate microfluidic channels, with each analyte measured in its own dedicated channel to eliminate cross-reactivity. Rather than true multiplexing, Ella performs up to four separate singleplex assays simultaneously from a single sample (~25 μL), combining the specificity of singleplex with the efficiency of multiplex, while providing full automation with built-in triplicate measurements for quality control. The Ella platform occupies an intermediate position in the AD biomarker assay landscape—offering enhanced sensitivity compared to conventional ELISA while providing practical advantages including full automation, rapid turnaround time (<90 min), and minimal sample volume requirements. However, like other high-sensitivity platforms, Ella remains primarily RUO for AD biomarker applications, lacking the regulatory clearance achieved by fully automated clinical platforms, such as Elecsys and Lumipulse. The correct interpretation of results requires precise knowledge of the assay used, as absolute biomarker concentrations differ substantially between platforms, necessitating platform-specific cutoff values [50,51].

### 3.3. Ultrasensitive Immunoassay Technologies

Ultrasensitive immunoassay platforms have revolutionized AD biomarker measurement by achieving sensitivity 100–1000 times higher than conventional ELISA. Single Molecule Array (Simoa) technology is the most widely adopted platform, utilizing a digital detection approach where individual enzyme-labeled molecules are isolated in femtoliter-volume wells (approximately 40 fL) [52,53]. The technology works by capturing target proteins on paramagnetic beads coated with capture antibodies, labelling them with enzyme-conjugated detection antibodies to form sandwich immunocomplexes, and then loading these beads into arrays of microscopic wells. When sealed with oil, each well containing an active enzyme generates a concentrated fluorescent signal that can be detected as a discrete “on” or “off” event, enabling digital counting of individual molecules at subfemtomolar concentrations [54]. This approach has enabled robust quantification of plasma Aβ42/40 ratio, phosphorylated tau species (p-tau181, p-tau217), neurofilament light chain (NfL), and GFAP with fully automated workflows suitable for clinical implementation.

### 3.4. Mass Spectrometry-Based Quantification

Immunoprecipitation-mass spectrometry (IP-MS) and targeted mass spectrometry have emerged as powerful hybrid technologies that combine the selectivity of antibody-based enrichment with the unparalleled specificity and accuracy of mass spectrometric detection, enabling measurement of low-abundance proteins that were previously considered unattainable. These technologies have fundamentally transformed the landscape of biomarker analysis, particularly in neurodegenerative disease research, where the detection of brain-derived proteins in peripheral blood requires extraordinary analytical sensitivity and specificity.

Immunoaffinity-mass spectrometry (IA-MS), also referred to as IP-MS, represents an emerging analytical genre that bridges the gap between traditional ligand binding assays (LBAs) and pure mass spectrometry approaches [55]. The technique combines target immunoaffinity enrichment with the use of stable isotope-labeled internal standards and MS detection, achieving high sensitivity while providing unparalleled specificity for the quantification of protein biomarkers [55,56]. In the SISCAPA (Stable Isotope Standards and Capture by Anti-Peptide Antibodies) variant, proteins are digested to component peptides using an enzyme such as trypsin, and one or more selected “proteotypic” peptides unique to the target protein are enriched using anti-peptide antibodies and measured as quantitative stoichiometric surrogates for protein concentration [57]. This hybrid approach achieves high sensitivity while providing unparalleled specificity for the quantification of protein biomarkers in fluids and tissues, overcoming the fundamental limitations inherent to antibody-based methods alone, including cross-reactivity, interference from heterophilic antibodies, and matrix effects.

Targeted mass spectrometry encompasses a family of acquisition strategies, including selected reaction monitoring (SRM), multiple reaction monitoring (MRM), and parallel reaction monitoring (PRM), that enable precise, reproducible quantification of predetermined protein targets in complex biological samples [58]. Unlike discovery-based proteomics approaches that aim to identify as many proteins as possible, targeted methods focus analytical resources on specific proteins of interest, achieving inherent reproducibility, unparalleled sensitivity (attomole level), and selectivity to efficiently differentiate isoforms, post-translational modifications, and mutated forms of proteins. The concept utilizes triple quadrupole (QqQ) mass analyzers for SRM/MRM or high-resolution/accurate-mass (HR/AM) instruments such as quadrupole-Orbitrap for PRM, with capacity for multiplexing approximately 100–200 proteins per analysis. In contrast to immunoaffinity-based workflows typically used in biological and clinical research, targeted MS is characterized by high selectivity, large capacity for multiplexing, and rapid, cost-effective transition from assay development to deployment.

The convergence of these technologies has proven particularly transformative for AD blood biomarker development. The challenge of measuring central nervous system-derived proteins such as Aβ peptides and p-tau in blood, where concentrations are 100–1000-fold lower than in CSF, required analytical platforms with extraordinary sensitivity and specificity. IP-MS methods, particularly those developed at Washington University (IP-MS-WashU) and Shimadzu (IP-MS-Shim), have demonstrated superior diagnostic performance compared to immunoassay-based approaches [59,60]. These MS-based methods also show the highest correlations with CSF biomarkers (r = 0.56–0.65), indicating superior reflection of brain pathology. Recent advances have streamlined IP-MS workflows, reducing antibody and sample volume requirements by approximately 75% while maintaining excellent precision (<10% variation), dilution linearity, and enhanced sensitivity, making these technologies increasingly practical for large-scale clinical implementation [61,62].

### 3.5. Targeted Proteomic Platforms

High-throughput targeted proteomic platforms have emerged as transformative technologies for biomarker discovery and validation in AD research, enabling simultaneous measurement of hundreds of proteins from minimal sample volumes [63,64]. These platforms bridge the gap between traditional single-analyte immunoassays and unbiased mass spectrometry-based proteomics, offering the multiplexing capacity needed to comprehensively profile the complex pathophysiology of neurodegenerative diseases while maintaining the sensitivity required for detecting low-abundance brain-derived proteins in peripheral blood.

Two major affinity-based platforms have driven enhanced sensitivity and throughput in AD proteome profiling: Olink and NULISA. Unlike pure discovery-phase methods such as untargeted mass spectrometry proteomics, which provide unbiased, hypothesis-free profiling of thousands of proteins across the entire proteome, NULISA and Olink employ pre-selected antibody panels targeting specific proteins of interest, thereby constraining the analytical scope to curated biomarker sets.

The Olink platform measures simultaneous hundreds of proteins using proximity extension assay (PEA) technology, while the NULISAseq CNS disease panel measures approximately 120 analytes and the inflammation panel approximately 250 analytes, both utilizing nucleic acid-linked immunosandwich assay technology with next-generation sequencing readout. This targeted approach fundamentally distinguishes these platforms from discovery methods, as the analyte selection is predetermined based on existing biological hypotheses rather than emerging from unbiased molecular profiling.

The PEA, developed by Olink Proteomics, represents an innovative technology that combines the specificity of antibody-based immunoassays with the sensitivity of quantitative PCR (qPCR) or next-generation sequencing (NGS). The technology relies on dual antibody recognition: two antibodies, each conjugated with unique DNA oligonucleotides, bind to different epitopes on the target protein [65]. When both antibodies bind in proximity to the same protein molecule, their DNA sequences hybridize and form a molecular barcode that is subsequently amplified by qPCR [66]. This dual-recognition requirement ensures high specificity and minimizes cross-reactivity commonly encountered in multiplexed immunoassays. PEA achieves femtomolar sensitivity and can measure proteins across a 5-log dynamic range. The Olink platform offers multiple panel configurations, from the Proseek Multiplex 96 × 96 panels measuring 92 proteins each to the Explore 3072 library offering approximately 3000 protein assays, with quantification achieved using either Fluidigm BioMark real-time PCR or Illumina NGS instruments. The platform requires only 1 microliter of sample per test and provides normalized protein expression (NPX) values on a log2 scale, representing relative rather than absolute quantification. The Olink Explore platform excels in detecting low-abundance proteins such as cytokines and chemokines and is particularly effective for challenging sample types like CSF, where protein content is typically low. The technology has been applied in more than 2000 peer-reviewed studies, as emerges from PubMed, demonstrating its potential for biomarker discovery across various diseases.

NULISA, developed by Alamar Biosciences, represents a next-generation proteomic platform that improves the sensitivity of traditional proximity ligation assays, achieving attomolar sensitivity, approximately 10,000-fold more sensitive than conventional PEA [67]. This dramatic improvement is achieved through a dual capture and release mechanism built into oligonucleotide-conjugated antibodies that suppresses assay background. The technology uses a sandwich immunoassay format where the target protein is captured, and upon binding of detection antibodies, barcoded reporter DNA is released and quantified by NGS [67]. NULISA enables highly multiplexed quantification of both low- and high-abundance proteins spanning a wide dynamic range by attenuating signals from abundant targets with unconjugated antibodies. The platform is fully automated, making broad proteomic analysis accessible for research and diagnostic applications.

Both platforms offer complementary strengths for AD biomarker research. Direct comparisons have demonstrated that NULISA provides superior detectability and dynamic range across various targets, while Olink offers a more extensive track record and broader panel options [67,68]. The multiplexing advantage of both platforms allows concurrent assessment of established biomarkers alongside novel candidates reflecting diverse pathophysiological processes, including tau and amyloid pathology, neuroinflammation, synaptic dysfunction, and neurodegeneration.

These targeted proteomic platforms are particularly valuable for biomarker discovery studies seeking to identify novel protein signatures associated with disease progression, differential diagnosis among neurodegenerative conditions, and prediction of clinical outcomes. The ability to measure condition-specific proteomic biomarkers while simultaneously assessing transdiagnostic markers like NfL enables comprehensive profiling that supports both mechanistic research and clinical translation. As these platforms continue to mature, standardization of preanalytical protocols, extension of validation across diverse populations, and integration with other omics data will be essential for realizing their full potential in transforming early AD diagnosis and enabling personalized interventions.

## 4. Clinical Phase

The transition of AD fluid biomarkers from research tools to clinical diagnostics represents a landmark achievement in the field, with several assays now receiving regulatory approval for clinical use in the United States and European countries. These approvals mark a paradigm shift in AD diagnosis, enabling scalable, accessible testing that can reduce reliance on costly positron emission tomography (PET) imaging by approximately 80–90% [69,70].

Clinical-phase methods prioritize automation, standardization, regulatory compliance, and scalability to enable routine diagnostic use. These methods must meet stringent analytical and clinical validation requirements for regulatory approval and clinical implementation. For diagnostic assays to be used in clinical care, robust and scalable in vitro diagnostic (IVD) assays approved by certifying bodies are needed. The transition from research-use-only assays and laboratory-developed tests to clinically validated IVDs requires navigating complex regulatory frameworks that vary across jurisdictions, with the United States Food and Drug Administration (FDA) and European regulatory authorities representing the two major routes for market authorization [9].

Lumipulse G (Fujirebio) and Roche Elecsys are the two leading fully automated immunoassay platforms for measuring AD biomarkers in both CSF and plasma.

The Lumipulse G system is a fully automated chemiluminescent enzyme immunoassay (CLEIA) platform that uses bead-based immunoassays to measure AD biomarkers including Aβ1-42, Aβ1-40, total tau, phosphorylated tau (p-tau181 and p-tau217), and biomarker ratios. The technology employs magnetic microparticle-based capture with chemiluminescent detection, enabling high-throughput processing on instruments such as the Lumipulse G600II and G1200.

The Elecsys system uses electrochemiluminescence immunoassay (ECLIA) technology on the cobas e 601/e 801 analyzers, which are already widely implemented in clinical chemistry laboratories worldwide. The platform measures CSF and plasma Aβ42, Aβ40, total tau, p-tau181, and more recently p-tau217, using ruthenium-labeled antibodies that generate electrochemiluminescent signals upon voltage application.

The regulatory approval of CSF and blood-based biomarkers for AD has progressed from early CE-IVD implementation in Europe to more recent U.S. FDA authorizations, reflecting increasing clinical validation and standardization (Table 1). CSF biomarkers were the first to achieve routine clinical use. The fully automated Lumipulse panel (Fujirebio) obtained CE-IVD marking in a stepwise manner, beginning with Aβ1-42 in January 2017 and total tau (tTau) on 16 July 2017, followed by the introduction of Aβ1-40 and phosphorylated tau (pTau181) in November 2018, thereby establishing a comprehensive CE-marked panel for clinical laboratories. These CSF assays are intended as adjunctive diagnostic tools in patients with cognitive impairment, enabling the in vivo assessment of core AD pathophysiological processes, with decreased Aβ42 or Aβ42/Aβ40 ratio reflecting amyloid plaque deposition and increased pTau181 and tTau indicating tau pathology and neurodegeneration. In the United States, CSF biomarkers were subsequently incorporated into FDA-authorized systems, most notably the Lumipulse G β-Amyloid Ratio (1-42/1-40), which received De Novo authorization on 4 May 2022, as an aid in identifying amyloid pathology in adults (≥55 years) presenting with cognitive impairment, with later extensions including tau biomarkers within the same platform framework. The Roche Elecsys CSF assays for Aβ1-42, pTau181, and tTau are CE-marked for clinical use in Europe and received U.S. FDA 510(k) clearance in 2022–2023 when used as biomarker ratios (pTau181/Aβ42 and tTau/Aβ42) in adults aged ≥55 years with cognitive impairment; these assays are intended as adjunctive tools to aid in the identification of AD pathology by reflecting amyloid deposition and tau-related neurodegeneration, whereas Aβ1-40 is not included in the FDA-cleared Elecsys CSF diagnostic system. They were introduced as CE-marked IVDs in Europe in the mid-to-late 2010s (approximately 2016–2018), with subsequent updates under the IVDR framework. Notably, the Elecsys Aβ1-40 CSF assay is also available as an IVD test in Europe and is primarily used to calculate the Aβ42/Aβ40 ratio, which improves the accuracy of amyloid assessment by correcting for interindividual variability in amyloid production. Importantly, unlike the stepwise CE-mark announcements seen with some other platforms, Roche Elecsys CSF assays were introduced as fully automated, standardized immunoassays already CE-marked upon commercial release, and are now broadly implemented across European clinical settings. Overall, the CE-marked Elecsys CSF biomarker panel is intended as an adjunctive diagnostic system to aid in the identification of AD pathology in patients with cognitive impairment, supporting early and more accurate diagnosis through the combined assessment of amyloid and tau pathophysiological processes.

More recently, blood-based biomarkers have achieved regulatory milestones, marking a shift toward minimally invasive testing. The Lumipulse G pTau217/β-Amyloid 1-42 plasma ratio was FDA-cleared on 16 May 2025, intended as an aid in the detection of amyloid pathology in cognitively impaired adults, while the Elecsys pTau181 plasma test received FDA clearance on 13 October 2025, with a complementary role as a rule-out test to exclude Alzheimer’s-related amyloid pathology in symptomatic individuals in primary care settings. Collectively, these biomarkers, whether CSF-based or blood-based, are not standalone diagnostic tests but are intended to support clinical decision-making, improving diagnostic accuracy, guiding patient selection for confirmatory imaging or therapy, and enabling earlier and more accessible detection of AD across different healthcare settings.

## 5. Discussion

This review highlights the remarkable evolution of AD fluid biomarker development from exploratory discovery platforms to clinically implemented diagnostic tools, illustrating both the scientific progress achieved and the persistent challenges that limit full translation into routine care. The shift toward a biological definition of AD, grounded in measurable molecular pathology rather than clinical phenotype alone, has fundamentally reshaped the diagnostic landscape. Fluid biomarkers, particularly Aβ42, t-tau, and p-tau, now form the backbone of this framework, enabling in vivo detection of core pathological processes. However, the journey from biomarker discovery to clinical implementation remains complex, with substantial attrition across the development pipeline.

A key theme emerging from this review is the critical importance of aligning analytical methodologies with the specific phase of biomarker development. Discovery-phase technologies, particularly MS-based proteomics, offer unparalleled breadth and hypothesis-free exploration, enabling identification of novel candidate biomarkers across diverse biological pathways. These approaches have been instrumental in expanding the repertoire of potential AD biomarkers beyond the traditional amyloid and tau axes, incorporating markers of neuroinflammation, synaptic dysfunction, and neurodegeneration. However, their complexity, cost, and limited scalability constrain their direct clinical applicability, reinforcing their primary role as discovery tools rather than diagnostic platforms.

The transition from discovery to research-phase validation introduces a shift toward targeted, reproducible, and scalable methods. Immunoassay-based platforms, including ELISA, ECL, microfluidic systems, and ultrasensitive technologies such as Simoa, have been central to this stage. These methods have enabled robust quantification of biomarkers in well-characterized cohorts and facilitated large-scale validation studies. Notably, ultrasensitive platforms have been transformative in enabling reliable detection of AD biomarkers in blood, overcoming the long-standing challenge of low analyte concentrations outside the central nervous system. This advancement represents a major step toward minimally invasive, scalable diagnostics.

Mass spectrometry-based quantification, particularly immunoprecipitation-MS, emerges as a highly promising approach that addresses several limitations of antibody-based assays. By combining immunoaffinity enrichment with highly specific molecular detection, these methods offer superior analytical specificity, reduced susceptibility to interference, and strong correlation with central pathology. Their demonstrated diagnostic accuracy for plasma biomarkers suggests that MS-based approaches may serve as reference methods for standardization, even if widespread clinical implementation remains limited by cost and technical complexity.

Targeted proteomic platforms such as Olink and NULISA represent an important intermediate between discovery and clinical application, enabling high-throughput, multiplexed analysis of predefined biomarker panels. These technologies are particularly valuable for capturing the multidimensional nature of AD pathology and identifying composite biomarker signatures that may outperform single-analyte approaches, particularly in the context of AD co-pathologies involving vascular injury, neuroinflammation, synaptic dysfunction, and concomitant neurodegenerative processes. Their ultrasensitive detection capabilities further support the characterization of disease heterogeneity and may facilitate improved patient stratification and monitoring across the AD continuum. However, their reliance on preselected targets inherently limits their exploratory capacity, and further validation is required to translate multiplex signatures into clinically actionable tools.

The clinical phase marks a significant milestone, with the introduction of fully automated, standardized immunoassay platforms such as Lumipulse and Elecsys. These systems address many of the limitations observed in earlier methods, including variability, scalability, and regulatory compliance. Their integration into clinical workflows, supported by regulatory approvals in both Europe and the United States, reflects a maturation of the field and a transition toward routine biomarker-guided diagnosis. Importantly, the emergence of blood-based biomarkers represents a paradigm shift, offering the potential for widespread screening, early detection, and improved access to diagnostic testing.

Nevertheless, several challenges remain before fluid biomarkers can be fully integrated into standard clinical practice. First, clinical utility, beyond diagnostic accuracy, must be clearly demonstrated. While biomarkers can reliably detect underlying pathology, evidence that their use improves patient outcomes, guides treatment decisions, or enhances cost-effectiveness is still limited. Second, preanalytical and analytical standardization across platforms and laboratories is essential to ensure reproducibility and comparability. The substantial inter- and intra-laboratory variability observed with traditional ELISA platforms underscores the importance of assay standardization and highlights why many early biomarker findings failed to translate into clinical practice. Even with newer technologies, differences in assay design, calibration, and detection principles result in platform-specific absolute values, necessitating careful interpretation and limiting cross-study comparability. This lack of harmonization continues to hamper the establishment of universal diagnostic thresholds. Third, implementation barriers such as cost, infrastructure requirements, and clinician adoption must be addressed, particularly in resource-limited settings.

Another important consideration is the evolving role of biomarkers in the context of emerging disease-modifying therapies. As therapeutic options expand, biomarkers will increasingly be used not only for diagnosis but also for patient stratification, treatment monitoring, and prognostication. This shift underscores the need for biomarkers that are not only accurate but also dynamic and responsive to therapeutic interventions.

Finally, this review underscores the importance of a “fit-for-purpose” approach in biomarker development. No single analytical platform is optimal across all phases; rather, the successful translation of biomarkers depends on the strategic integration of complementary technologies, each suited to specific stages of development. Future progress will likely depend on continued collaboration across disciplines, standardization initiatives, and the integration of multi-omics data to capture the complexity of AD pathophysiology.

In conclusion, while substantial progress has been made in the development and implementation of fluid biomarkers for AD, significant gaps remain in achieving full clinical translation. Addressing these challenges will be critical for realizing the potential of biomarkers to enable earlier diagnosis, improve patient outcomes, and support the development of precision medicine approaches in AD.

## Figures and Tables

**Table 1:** Regulatory approval of CSF and blood-based biomarkers for AD.

Platform	Sample Type	Biomarker	Regulatory Status (Year Approval)	Clinical Use
Fujirebio Lumipulse G	Plasma	p-tau217/Aβ42 ratio	FDA-cleared (2025)	Rule-in and rule-out AD pathology in symptomatic adults ≥ 55 years
	CSF	Aβ42, Aβ40, pTau181, tTau	FDA-approved (2022)	Aid in the diagnosis of AD in adult patients ≥ 55 years presenting with cognitive impairment
			CE-marked (2018)	Aid in diagnosis of patients with cognitive impairment who are being evaluated for AD and other causes of cognitive decline.
Roche Elecsys	Plasma	p-tau181	FDA-approved (2025)	Aid in the initial assessment for AD and other causes of cognitive decline in patients ages 55 and older in the primary-care setting
			CE-marked (2025)	Rule-out test only for AD in symptomatic adults ≥ 55 years
	CSF	pTau181/Aβ42 and tTau/Aβ42	FDA-approved (2022–2023)	Aid in the identification of AD pathology in adults aged ≥55 years with cognitive impairment
		Aβ42, Aβ40, pTau181, and tTau	CE-marked (2016–2018)	Aid in the diagnosis of AD in adult patients with cognitive impairment

AD, Alzheimer’s disease; FDA, Food and Drug Administration; CE, Conformité Européenne; CSF, Cerebrospinal fluid; Aβ42, Amyloid-beta 42; Aβ40, Amyloid-beta 40; pTau181, Phosphorylated tau at threonine 181; p-tau181, Phosphorylated tau 181; p-tau217, Phosphorylated tau 217; tTau, Total tau; AD pathology, Alzheimer’s disease pathology.

## Data Availability

No new data were created or analyzed in this study. Data sharing is not applicable to this article.
